# Somatization in patients with predominant diarrhoea irritable bowel syndrome: the role of the intestinal barrier function and integrity

**DOI:** 10.1186/s12876-021-01820-7

**Published:** 2021-05-22

**Authors:** Laura Prospero, Giuseppe Riezzo, Michele Linsalata, Antonella Orlando, Benedetta D’Attoma, Marta Di Masi, Manuela Martulli, Francesco Russo

**Affiliations:** 1 Laboratory of Nutritional Pathophysiology, National Institute of Gastroenterology “S. de Bellis” Research Hospital, Via Turi 27, 70013 Castellana Grotte, BA Italy; 2 Scientific Direction, National Institute of Gastroenterology “S. de Bellis” Research Hospital, 70013 Castellana Grotte, BA Italy

**Keywords:** Dysbiosis, Inflammation, Intestinal permeability, Irritable bowel syndrome, Somatization, Symptom questionnaire

## Abstract

**Background:**

Irritable bowel syndrome (IBS) is characterised by gastrointestinal (GI) and psychological symptoms (e.g., depression, anxiety, and somatization). Depression and anxiety, but not somatization, have already been associated with altered intestinal barrier function, increased LPS, and dysbiosis. The study aimed to investigate the possible link between somatization and intestinal barrier in IBS with diarrhoea (IBS-D) patients.

**Methods:**

Forty-seven IBS-D patients were classified as having low somatization (LS = 19) or high somatization (HS = 28) according to the Symptom Checklist-90-Revised (SCL-90-R), (cut-off score = 63). The IBS Severity Scoring System (IBS-SSS) and the Gastrointestinal Symptom Rating Scale (GSRS) questionnaires were administered to evaluate GI symptoms. The intestinal barrier function was studied by the lactulose/mannitol absorption test, faecal and serum zonulin, serum intestinal fatty-acid binding protein, and diamine oxidase. Inflammation was assessed by assaying serum Interleukins (IL-6, IL-8, IL-10), and tumour necrosis factor-α. Dysbiosis was assessed by the urinary concentrations of indole and skatole and serum lipopolysaccharide (LPS). All data were analysed using a non-parametric test.

**Results:**

The GI symptoms profiles were significantly more severe, both as a single symptom and as clusters of IBS-SSS and GSRS, in HS than LS patients. This finding was associated with impaired small intestinal permeability and increased faecal zonulin levels. Besides, HS patients showed significantly higher IL-8 and lowered IL-10 concentrations than LS patients. Lastly, circulating LPS levels and the urinary concentrations of indole were higher in HS than LS ones, suggesting a more pronounced imbalance of the small intestine in the former patients.

**Conclusions:**

IBS is a multifactorial disorder needing complete clinical, psychological, and biochemical evaluations.

*Trial registration*: https://clinicaltrials.gov/ct2/show/NCT03423069.

## Background

Irritable bowel syndrome (IBS) is a widespread disease with a high prevalence worldwide. Based on the gastrointestinal (GI) symptom profiles encoded by the Rome criteria [1], four types of IBS can be distinguished: IBS with prevalent diarrhoea (IBS-D), IBS with prevalent constipation (IBS-C), alternate/mixed IBS (IBS-M), and unclassified IBS (IBS-U).

Apart from GI symptoms, IBS patients may suffer from depression and anxiety [2]. At the same time, these psychological disorders, together with stress, are positively correlated with IBS symptoms [3]. In this regard, IBS is considered a functional somatic syndrome as it is mainly characterised by subjective symptoms, suffering, and disability [4]. So, it is now suggested that a correct IBS management should consider both GI symptoms and the psychological profile [5].

The term somatization refers to “a tendency to experience and communicate psychological distress in the form of somatic symptoms seeking medical help for them” [6]. IBS patients score higher on somatization than healthy controls [7], but lower than patients with somatoform disorders [8]. To a certain extent, somatization could be considered more representative than other indicators of how psychological disorders can generate or exacerbate IBS symptoms [9, 10]. Somatization disorder and somatoform disorders are currently merged under the definition of somatic symptom disorder (SSD) [11]. The SSD and the functional GI diseases mostly coexist [12], ranging from 15 to 48% in IBS patients [13, 14]. This evidence is of particular interest considering the close relationship between pain and somatization in patients with functional diseases [15], including those with IBS [16].

In recent years, the pathophysiology of IBS-D has proven to be tightly linked to alterations of the small intestinal permeability (s-IP) and the associated “minimal inflammation” [17]. The dysfunctional intestinal barrier, mainly in the upper gut, could be the origin or consequence of persistent low-grade immune activation in IBS and inflammatory bowel disease (IBD) [18]. Increased biomarkers of the intestinal barrier integrity associated with increased lipopolysaccharide (LPS) and dysbiosis have been already demonstrated in patients with anxiety or depression [19] but little information, if any, is available about the relationship between somatization and intestinal permeability in IBS-D.

As a biochemical counterpart, circulating concentrations of GI peptides, markers of the serotonin pathways [20, 21], inflammatory interleukins (IL), i.e. IL-6, IL-10, tumour necrosis factor (TNF)-α, LPS [22–24], along with stress markers [25, 26] have been demonstrated to be higher in IBS patients than normal subjects. Our group has already published data illustrating the state of health of the intestinal barrier in IBS-D patients compared to healthy controls [27]; thus, the present study aimed to verify whether somatization could be associated to the intestinal permeability as well as other biochemical determinants related to the intestinal barrier function. In this framework, IBS-D patients were enrolled to (a) assess the somatization levels identified administering the Symptom Checklist-90-Revised— (SCL-90-R); (b) investigate the GI symptom profile (by administering the IBS Severity Scoring System—IBS-SSS and the Gastrointestinal Symptom Rating Scale—GSRS); (c) study the role of the intestinal barrier by evaluating: the urinary Lactulose/Mannitol (La/Ma) ratio as markers of s-IP, the urinary sucrose (Su) as a marker of gastroduodenal permeability, and the biomarkers of GI barrier function and integrity (serum and faecal zonulin, serum intestinal fatty-acid binding protein—I-FABP, and serum diamine oxidase—DAO). The pro-inflammatory IL-6 and IL-8and anti-inflammatory IL-10 were also assessed. Additionally, LPS and the urinary indole and skatole as markers of intestinal dysbiosis were evaluated.

## Methods

### Patient recruitment

Patients suffering from IBS-D according to Rome IV criteria [1] were recruited from January 2018 to May 2020 from among the outpatients of the National Institute of Gastroenterology “S. de Bellis” Research Hospital, Castellana Grotte (Bari), Italy.

All patients completed validated psychological and symptom questionnaires (see below). They underwent a physical examination, a blood withdrawal (for whole blood count, liver function tests, C-reactive protein, thyroid function test), stool culture, stool examination for parasites, faecal occult blood test. The availability of a recent gastroscopy and colonoscopy to avoid the enrolment of patients with organic diseases was also requested.

The inclusion criteria were as follows: (a) age more than 18 years; (b) a symptom profile resembling IBS-D (with active symptoms for at least two weeks) and a stool pattern as described by Schmulson et al*.* [28]; (c) a minimum average of 3.0 on the seven-point Likert scale of the GSRS composite symptom [29]; (d) a diet without any restriction on eating and drinking (in particular, no previous period of gluten-free diet before examination). Age, body mass index (BMI), alcohol intake, smoking, and medication use were checked to obtain a homogeneous group of IBS-D.

Exclusion criteria included: pregnancy, constipation, giardiasis, post-infectious IBS, hepatic, renal, or cardiovascular disease, metabolic and endocrine disorders, a history of SSRIs and other antidepressant therapy, fever, intense physical activity, previous abdominal surgery, a history of malignancy, secondary causes of intestinal atrophy, no consumption of drugs for treating IBS in the last two weeks before evaluation, antibiotic therapy or probiotic agents, and other drugs known to cause abdominal pain. For excluding celiac disease, a combination of tissue transglutaminase (tTG) and anti-endomysium (EMA) antibodies was used. Additionally, only the HLA-DQ2/HLADQ8-negative/negative IBS-D patients were recruited in this study to avoid the possible presence of gluten-sensitive diarrhoea without celiac disease that has been observed in IBS patients positive for HLA-DQ2 or HLA-DQ8 [30].

Healthy individuals were recruited from among the administrative staff of our institute and the students attending the Nurse School of the University of Bari as controls (HC). They denied having dyspepsia or other GI diseases, metabolic, endocrine, or immunological diseases and did not take any medication. Information on the health status of participants was obtained by an interview on the current diet, lifestyle, medical history, and, lastly, by physical examination. As criteria of admission, EMA and tTG had to be negative. Besides, blood glucose, HbA1c, lipid profile, and blood pressure had to be within the normal range of values. The absence of major psychiatric disorders, cancer, and pregnancy were also inclusion criteria.

Female patients/HC were examined during the follicular phase of the menstrual cycle. All the subjects were compliant and were willing to participate in the study. Informed consent was obtained from all the participants for blood testing and clinical data collection.

This study was part of a research project approved by the local Scientific and Ethics Committee of IRCCS “Saverio de Bellis”, Castellana Grotte, Bari, Italy, and it was registered on clinicaltrials.gov (https://clinicaltrials.gov/ct2/show/NCT03423069).

The research project has been performed in accordance with the ethical standards as laid down in the 1964 Declaration of Helsinki and its later amendments or comparable ethics.

### Psychological questionnaire

#### Symptom checklist-90-Revised (SCL-90-R)

The Symptom Checklist-90- Revised (SCL-90-R) is one of the best known and most used self-report measures in the psychopathological field [31]. SCL-90-R evaluates a broad spectrum of psychopathological symptoms, namely nine primary symptom dimensions and three global indices. We have considered only the Global Severity Index (GSI), the best indicator of the current intensity of psychic distress perceived by the subject. The raw scores were transformed into T scores, and the T scores equal to or above 63 were considered indicative of clinically significant symptomatology [32, 33].

### Symptom assessment

#### IBS severity scoring system (IBS-SSS)

The symptom profile was investigated by administering a validated GI symptoms questionnaire, the IBS-SSS [34, 35]. IBS‐SSS is a five‐item questionnaire measuring frequency and intensity of abdominal pain, the severity of abdominal distension, dissatisfaction with bowel habits, and the interference of IBS with daily life, scoring from 0 to 500. We applied the widely used cut‐off of IBS‐SSS scores to evaluate the severity of IBS: < 175 for mild IBS, 175‐300 for moderate IBS, and > 300 for severe IBS.

#### Gastrointestinal symptom rating scale (GSRS)

GSRS is a validated GI questionnaire that utilises a 7-level Likert scale (1–7), based on the intensity and frequency of GI symptoms experienced during the previous seven days. A higher score represents the main symptoms complained about by the patients. The 7-level scores were then merged to obtain a four-level score of intensity/frequency: absent, mild, moderate, and severe. The combination scores among the GSRS items identified four clusters: (a) “Abdominal pain syndrome” collects abdominal pain, gastric hunger pain, and nausea scores; (b) “Dyspepsia syndrome” collects halitosis, heartburn, and regurgitation scores; (c) “Indigestion syndrome” collects abdominal distension, borborygmi, burping, and flatulence scores; (d) “Diarrhoea syndrome” collects increased frequency of evacuation, loose stools, and urgent need to defecate scores [36].

### Sugar absorption test

All the participants in the study underwent s-IP evaluation by sugar absorption test after fasting overnight. Pre-test urine was collected in our laboratory to check for the possible presence of endogenous sugars. Then subjects drank a sugar test solution containing 10 g of lactulose, 5 g of mannitol, and 40 g of sucrose in a volume of 100 ml. Urine samples were collected up to 5 h after administration. A 1-ml volume of 20% (w/v) chlorohexidine was added to each collection as a preservative regardless of the final volumes. The total urine volumes from individuals were measured and recorded. After thoroughly mixing, a portion of 2 ml was taken and stored at − 80 °C until analysed. The detection and measurement of the three sugar probes, La, Ma, and Su, in urine were performed by chromatographic analysis, as described previously by our group [37]. In brief, high-performance anion-exchange chromatography coupled with pulsed amperometric detection was performed on a Dionex Model ICS-5000 with a gold working electrode and a 25 μl peek sample loop (Dionex Corp., Sunnyvale. California, USA). The carbohydrate separation was performed using a Carbopac PA-10 pellicular anion-exchange resin connected to a Carbopac PA-10 guard column (Thermofisher Scientific, Waltham, Massachusetts, USA) at 30 °C. The samples were eluted with 50 mmol/l NaOH at a 1 ml/min flow rate. The percentage of ingested La (%La), Ma (%Ma), and Su (%Su) were evaluated in urine, and the La/Ma ratio was calculated for each sample. Patients with a La/Ma ratio higher than 0.030 were considered as having an altered s-IP [27].

### Biochemical analyses

The blind coded samples of a whole blood sample were taken from each IBS-D patient, after 12 h of fasting, by venous puncture. Blood samples were collected in vacutainer tubes containing ethylene–diamine–tetra-acetic acid (EDTA-K2) anticoagulant. EDTA tubes were centrifuged at 2000 × g for 15 min, and blood samples were stored at − 20 °C until the assay was performed. Raw stool samples from the IBS-D patients were frozen and stored at − 80 °C within 12 h after the sampling. Before the laboratory analysis, stool samples were thawed, and mechanical homogenisation was performed using an inoculation loop. The faecal sample preparation kit (Immunodiagnostik AG, Bensheim, Germany) to prepare faecal eluates was used.

Serum and faecal zonulin were assayed by an enzyme-linked immunosorbent assay (ELISA) kit (Immunodiagnostik AG, Bensheim, Germany). Serum concentrations of I-FABP and DAO were evaluated by ELISA (Thermo Fisher Scientific, Waltham, Massachusetts and Cloud-Clone Corp, Houston, TX, USA, respectively).

Serum concentrations of IL-6, IL-8, IL-10, and TNF-α were measured using commercially available ELISA kits (BD Biosciences, Milan, Italy). LPS was assayed using an ELISA kit by Cloud-Clone Corp (Katy, TX, USA).

### Indole and Skatole evaluation

All patients collected a sample of urine in the morning. A standard colorimetric assay kit (Indican Assay Kit, ABNova Corporation, Taipei, Taiwan) was used according to the manufacturer's procedures for urinary indole determination. The detection and measurement of skatole in urine were performed by the 3-methylindole kit (Eureka Lab Division, Chiaravalle, AN, Italy) on a Thermo Scientific model Dionex high-performance liquid chromatography (HPLC) system consisting of an UltiMate 3000 pump and a Rheodyne injector with a 20-µL loop (Sunnyvale, CA, USA). Samples, calibrators, and quality controls were prepared according to the manufacturer’s instructions. In detail, 950 μL of buffer reagent and 20 μL of the internal standard were added to 50 μL of a urinary sample. After vortexing, 20 μL of urine samples were injected into the HPLC system. A Poroshell 120 EC-C18 column (2.7 µm. 50 × 4.6 mm; Agilent, Santa Clara, CA, USA) and a mobile phase flow rate of 1.0 ml/min were used for the skatole separation. The sample run was 15 min, and spectrofluorimetric detector wavelengths were set at 280 nm (excitation) and 360 nm (emission). Urinary indole and skatole values higher than 20 mg/l and 20 ng/l are considered indices of fermentative and putrefactive dysbiosis, respectively [38].

### Statistical analysis

All results are expressed as mean ± SD and median and range for continuous or discrete variables, respectively. To avoid the assumption of the normal distribution, the Mann–Whitney rank-sum test was used to assess differences between the groups. Chi-square/Fisher’s exact test was used to compare dichotomous variables. A specific statistical package for exact non-parametric inference (2005 Stata Statistical Software Release 9; Stata Corp., College Station, TX, USA) was used. All the differences were considered significant at a 5% level.

## Results

### Study group description

**Fig. 1:**
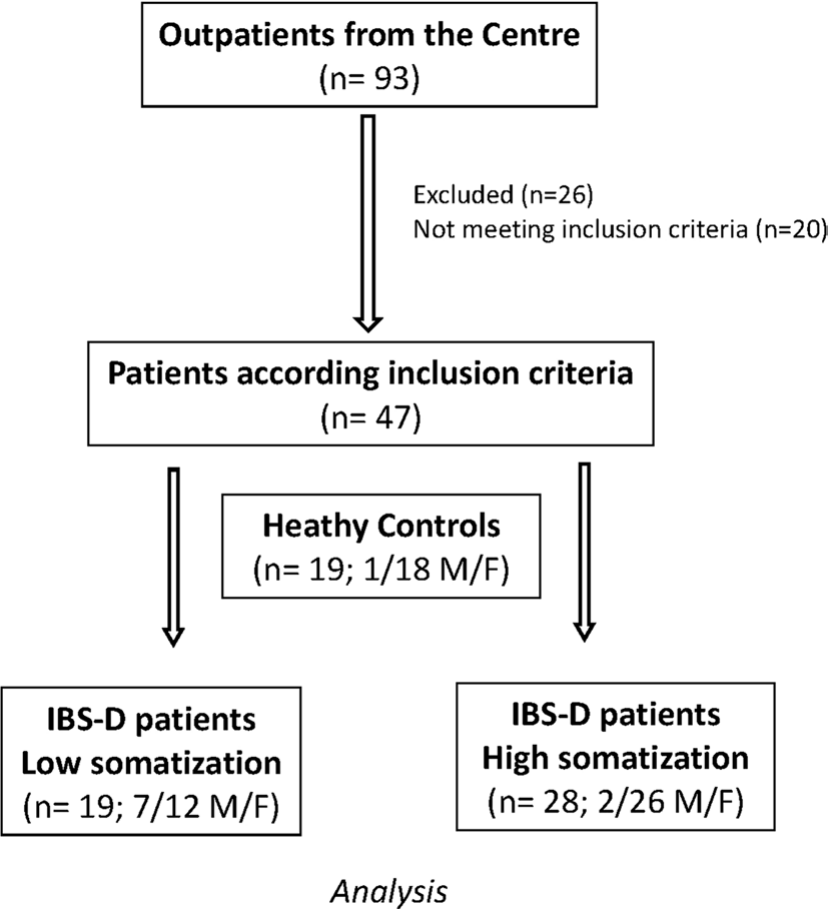
The flow chart of the patients

Figure 1 shows the flow chart of the number of patients. Forty-seven IBS-D patients (9 men and 38 women; mean age = 42.8 ± 10.3 yrs.; BMI = 24.8 ± 4.6) and 19 HC subjects (1 man and 18 women; mean age = 32.5 ± 14.1 yrs.; BMI = 24.5 ± 6.1) completed the study. The difference between the mean ages of IBS and HC was statistically significant (*P* = 0.004) since the HC group consisted mostly of young subjects who guaranteed a lower probability of somatization.

Table 1 describes the psychological profile of IBS-D patients and HC that participated in the study. As expected, there is evidence of higher levels of depression, anxiety and somatization in IBS-D patients than in HC.

**Table 1:** Mean scores of SCL-90 R domains in subject with and without IBS-D

	IBS-D (n = 47)	HC (n = 19)	*P*
Global Severity Index	66.66 ± 21.59	49.42 ± 11.88	0.002
Somatization	68.19 ± 17.88	49.31 ± 9.51	< 0.001
Obsessive–compulsive	62.48 ± 19.43	53.05 ± 14.10	0.06
Interpersonal sensitivity	58.19 ± 18.85	51.05 ± 10.68	0.23
Depression	65.72 ± 21.57	49.57 ± 10.38	0.004
Anxiety	64.31 ± 21.98	47.10 ± 10.56	0.001
Hostility	55.85 ± 12.98	47.89 ± 5.97	0.02
Phobic anxiety	56.76 ± 20.23	50.63 ± 9.83	0.38
Paranoid ideation	56.76 ± 20.23	50.63 ± 9.83	0.18
Psychoticism	60.21 ± 20.82	50.21 ± 13.42	0.05

Data are expressed as means ± SD. *P*-Value was determined by Mann–Whitney test rank-sum test; differences were considered significant at  *P *< 0.05. IBS-D, Irritable bowel syndrome with diarrhoea; HC, Healthy controls

Grouping the IBS-D patients according to somatization level, 19 IBS-D patients (7 men and 12 women; mean age = 41.84 ± 10.13 yrs., BMI 25.17 ± 4.07) had low somatization level (LS subgroup), whereas 28 IBS-D patients (2 men and 26 women; mean age = 43.39 ± 10.67 yrs.; BMI 25.07 ± 5.06) had high somatization level (HS subgroup). No difference was reported regarding the anthropometric characteristic between the two groups.

### GI symptom profile

As for the GI symptom profile, the total IBS-SSS score was similar in the two groups, while the individual items “Abdominal pain severity” (*P* = 0.04) and “Abdominal pain duration” (*P* = 0.03) were significantly higher in the HS subgroup (Table 2).

**Table 2:** Item scores of IBS-SSS in IBS-D patients with low (LS) and high scores (HS) of somatization

	LS (n = 19)	HS (n = 28)	*P*
Abdominal pain severity	38.68 ± 23.85	53.03 ± 22.74	0.03
Abdominal pain duration (days)	33.15 ± 28.09	50.71 ± 27.47	0.03
Abdominal distension severity	53.31 ± 27.12	58.14 ± 19.63	0.54
Bowel habit satisfaction	70.10 ± 20.78	65.85 ± 23.99	0.73
Life disruption	60.00 ± 20.27	57.89 ± 23.12	0.88
Total score	255.26 ± 78.11	285.64 ± 85.90	0.16

Data are expressed as means ± SD. *P*-Value was determined by Mann–Whitney test rank-sum test; differences were considered significant at  *P*< 0.05. LS, Low somatization level; cut-off score < 63; HS, High somatization level; cut-off score ≥ 63

Regarding the GSRS questionnaire, the symptom scores were significantly higher in the HS subgroup than in the LS one. In particular, the GSRS clusters “Abdominal pain syndrome” and “Indigestion syndrome” were significantly higher in the HS patients. The individual items of the GSRS questionnaire, i.e. "Abdominal pain" (*P* < 0.001), "Borborygmus" (*P* = 0.02) and "Abdominal distension" (*P* = 0.03) were significantly higher in the HS subgroup, while the items relating to the bowel habit did not show any significant differences between the two subgroups (Tables 3 and 4).

**Table 3:** Scale scores of GSRS in IBS-D patients with low (LS) and high scores (HS) of somatization (Subscale of SCL-90-R)

	LS (n = 19)	HS (n = 28)	*P*
*Clusters*			
Abdominal pain syndrome	5.00 [3.00–8.00]	7.00 [4.00–9.27]	0.003
Dyspepsia syndrome	6.00 [3.00–9.95]	6.00 [3.40–9.60]	0.85
Indigestion syndrome	10.00 [4.00–12.00]	10.25 [8.00–14.55]	0.04
Diarrhoea syndrome	6.00 [4.00–9.00]	7.00 [5.00–9.50]	0.38
Total score	33.00 [18.00–38.00]	36.50 [27.90–44.55]	0.01

Data are expressed as Medians and Range. *P*-Value was determined by Mann–Whitney test rank-sum test; differences were considered significant at *P* < 0.05. LS, Low somatization level; cut-off score < 63. HS, High somatization level; cut-off score ≥ 63

**Table 4:** Item scores of GSRS in IBS-D patients with low (LS) and high scores (HS) of somatization

	LS (n = 19)	HS (n = 28)	* P*
*Single items*			
Halitosis	2.00 [1.00–4.00]	2.00 [1.00–4.00]	0.60
Abdominal pain	2.00 [1.00–3.00]	3.00 [2.00–3.77]	< 0.001
Heartburn	1.00 [1.00–3.00]	2.00 [1.00–3.55]	0.37
Acid regurgitation	2.00 [1.00–3.00]	2.00 [1.00–3.55]	0.08
Sucking sensation in the epigastrium	2.00 [1.00–3.00]	2.00 [1.00–3.55]	0.20
Nausea and vomiting	1.00 [1.00–3.00]	1.75 [1.00–3.00]	0.14
Borborygmus	2.00 [1.00–3.00]	3.00 [2.00–4.00]	0.02
Abdominal distension	3.00 [1.00–4.00]	3.00 [2.00–4.00]	0.03
Eructation	2.00 [1.00–3.00]	2.00 [1.00–4.00]	0.87
Increased flatus	3.00 [1.00–3.00]	3.00 [2.00–4.00]	0.14
Decreased passage of stools	1.00[1.00–4.00]	1.00 [1.00–4.00]	0.51
Increased passage of stools	2.00 [1.00–3.00]	2.00 [1.00–3.00]	0.97
Loose stools	2.00 [1.00–3.00]	2.00 [1.45–3.77]	0.42
Hard stools	1.00 [1.00–2.00]	1.00 [1.00–4.00]	0.05
Urgent need for defecation	2.00 [1.00–3.00]	3.00[1.45–3.55]	0.30
Feeling of incomplete evacuation	2.00 [1.00–3.00]	2.50 [1.00–4.00]	0.12

Data are expressed as Medians and Range. *P*-Value was determined by Mann–Whitney rank-sum test; differences were considered significant at  *P* < 0.05. LS, Low somatization level; cut-off score < 63. HS, High somatization level; cut-off score ≥ 63

### The small intestinal permeability (s-IP)

**Fig. 2:**
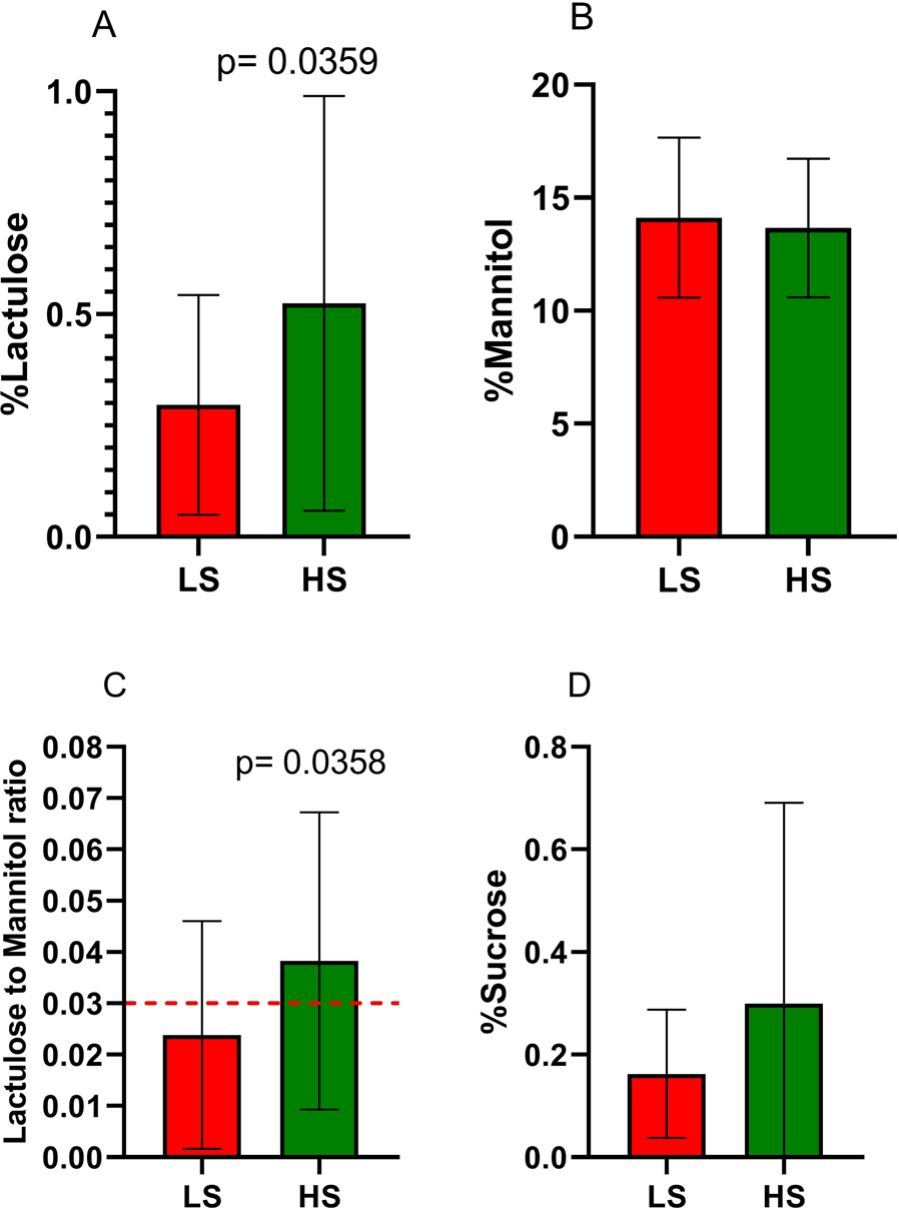
Urinary markers of gastrointestinal permeabilityin IBS-D with low (LS) and high (HS) somatization. Panel A = %Lactulose (La). Panel B = %Mannitol (Ma). Panel C = La/Ma ratio. Panel D = %Sucrose (Su). The La/Ma ratio was significantly higher in HS patients than in LS ones (P = 0.0358). The red dot line indicates the cut-off level. Data are expressed as means ± SD and evaluated by the Mann-Whitney rank-sum test

The s-IP in IBS-D patients categorised according to the somatization levels is reported in Fig. 2. Significantly higher percentages of lactulose urinary excretion were expressed in the HS subgroup compared to LS one (0.52 ± 0.088 vs 0.30 ± 0.057, *P* = 0.036). By opposite, the urinary percentages of mannitol were similar in the two subgroups (14 ± 0.81 vs 14 ± 0.58; *P* = 0.68). Consequently, the La/Ma ratio was significantly higher in the HS patients than LS ones (0.038 ± 0.03 vs 0.024 ± 0.022; *P* = 0.036). As for the gastroduodenal permeability, the sucrose excretion in urine was not significantly different between the two subgroups, although a higher percentage of excretion was reported in the HS compared to LS (0.30 ± 0.39 vs 0.16 ± 0.13; *P* = 0.99). Noteworthy, in the subgroup of HS patients, 16 out of 28 patients (57%) showed altered intestinal permeability (La/Ma > 0.030), whereas 3 out of the 19 LS patients (15.8%) had an alteration in the intestinal permeability at the sugar absorption test. The difference was significant at the Fisher exact test (*P* = 0.006).

### The markers of the intestinal barrier function and integrity and the indices of inflammation

The markers of intestinal barrier function and integrity (faecal and serum zonulin, I-FABP, and DAO) together with the indices of inflammation (IL-6, IL-8, IL-10, and TNF-α) are reported in Table 5. Faecal zonulin concentrations were significantly higher in the HS group than the LS one (*P* = 0.035), while serum zonulin did not. Regarding the inflammation indices, the pro-inflammatory IL-8 levels were significantly higher (*P* = 0.007), and the anti-inflammatory IL-10 concentrations were significantly (*P* = 0.02) lower in the HS patients than LS ones.

**Table 5:** Mean serum concentrations of biochemical parameters in IBS-D patients with low (LS) and high scores (HS) of somatization (Subscale of SCL-90-R)

	LS (n = 19)	HS (n = 28)	*P*
IFABP-2 (ng/ml)	3.76 ± 5.94	2.97 ± 2.96	0.19
DAO (ng/ml)	36.10 ± 5.12	35.53 ± 4.32	0.57
Faecal Zonulin (ng/ml)	127.0 ± 67.0	180.0 ± 90.0	0.035
Serum Zonulin (ng/ml)	29.0 ± 7.1	29.0 ± 5.5	0.81
IL-6 (pg/ml)	5.76 ± 1.61	6.83 ± 7.52	0.97
IL-8 (pg/ml)	3.71 ± 0,60	5.17 ± 2.88	0.007
IL-10 (pg/ml)	3.44 ± 0.78	2.94 ± 0.28	0.02
TNF-α(pg/ml)	3.92 ± 1.22	3.57 ± 0.50	0.14

Data are expressed as means ± SD. *P*-value was determined by Mann–Whitney rank-sum test; differences were considered significant at *P* < 0.05. LS, Low somatization level; cut-off score < 63. HS, High somatization level; cut-off score ≥ 63

### The markers of intestinal dysbiosis and bacterial translocation

All the IBS-D patients showed indole concentrations higher than the cut-off level (< 20 mg/l), signifying the presence of a fermentative dysbiosis. Besides, when the patients were categorised according to somatization levels, a significant difference (*P* = 0.02) in the indole concentrations was observed between LS patients (51.56 ± 31.96 mg/l) and HS ones (81.53 ± 46.13 mg/l).

On the contrary, the skatole concentrations in urine were within the limit of the normal range (below 20 µl/l) in both the LS and HS groups (7.46 ± 8.54 µg/L vs 11.00 ± 10.51 µg/L; *P* = 0.25), suggesting the absence of putrefactive dysbiosis in IBS-D patients, irrespective of somatization.

Finally, the LPS concentrations were significantly (*P* = 0.04) higher (0.05 ± 0.01 ng/ml vs 0.03 ± 0.01 ng/ml) in the HS than the LS group, suggesting an increased passage of microbial components in the former group.

## Discussion

The present results confirm the close relationship between IBS symptoms profile, psychological disorders, and alterations in the intestinal barrier's integrity and function. In the IBS-D patients with HS, the GI symptoms were significantly more severe, both as a single symptom and as a cluster. This finding was associated with impaired intestinal permeability, inflammation, and dysbiosis in the small intestine.

To analyse the psychological profile of IBS patients, the SCL-90-R was administered. IBS-D patients showed scores higher than the cut-off in GSI, somatization, depression, and anxiety compared to HC [32, 33]. Overall, these findings agree with data in the literature about the participation of emotional and psychological aspects in IBS-D patients.

There is still an open debate on the pathophysiological basis of IBS, and a double aetiology, biological and psychological, has been proposed.

In a recent paper by Soncini et al. [39] aimed at describing new diagnostic tools and treatments for IBS patients by Italian gastroenterologists, the authors underlined the importance of quality of life and psychological involvement as factors able to affect the symptom severity and the possible use of psychotherapy for management of the disease.

Another study on a large cohort of Italian IBS patients analysed the multifactorial pathophysiology of IBS [40], reporting a high rate of psychiatric comorbidities in these individuals. The authors found that one-third of their patients had a diagnosis of mental disorder. Compared to this report, our patients did not suffer from psychiatric comorbidities, even if approximately 60% of them showed high levels of somatization.

Based on the concept of the amplification of the gut’s sensation due to somatization, it has been postulated that IBS patients and, more in general, patients with functional GI disorders and a high level of somatization may show a more severe symptom profile than patients without somatization. In particular, more intense and frequent regurgitation, bloating and abdominal pain, discomfort, and distension were shown by these patients [15, 41]. Consistent with this evidence, in our cohort of patients, the IBS-SSS and GSRS scores were significantly different between HS and LS subgroups. The items of the IBS-SSS questionnaire "Abdominal pain severity" and "Abdominal pain duration", and the single items of the GSRS questionnaire "Abdominal pain", "Abdominal distension", and "Borborygmus" were significantly higher in HS than LS patients. The "Abdominal pain syndrome” and “Indigestion syndrome " clusters were also significantly higher in HS than LS groups. In contrast, bowel habit items were not significantly different between the two groups.

These findings derive from validated questionnaires generally used to evaluate the symptom profile of IBS patients. The IBS-SSS and the GSRS were administered to the IBS-D patients of our study, as the former focuses only on abdominal pain and distension, whist the GSRS evaluates a broader range of GI symptoms [29, 34].

Overall, these data highlight how high somatization can negatively affect several GI symptoms in IBS-D, confirming once more the need for a psychological evaluation and management of these patients to obtain an adequate diagnostic and therapeutic framework.

Like other psychiatric and functional disorders, somatization lacks biological markers being diagnosed on subjectively reported symptoms by administering multiple psychological questionnaires. However, there is a close relationship between “leaky gut”, intestinal inflammation, and dysbiosis involving the "brain-gut-microbiota" axis or the bidirectional communication between the enteric nervous system and the central nervous system [42].

As already reported in previous papers [19, 27, 36], alterations in the intestinal barrier can be associated with an altered psychological profile [43]. In our study, 57% of HS patients had an altered intestinal permeability, while only 16% of the LS patients suffered from altered s-IP. Besides, faecal zonulin concentrations were higher in HS patients than LS ones, supporting the concept of impaired intestinal permeability in the former group. Faecal zonulin is considered a more specific marker for impaired intestinal permeability  due to its local action at the intestinal level in the case of a weakened intestinal barrier compared to serum zonulin [44].

Data in the literature have shown that patients with major depression show increased expression of pro-inflammatory cytokines and their receptors and increased concentrations of acute-phase markers, chemokines, and soluble adhesion molecules in peripheral blood and cerebrospinal fluid [45]. Peripheral blood gene expression profiles revealed overexpression of IL-6-induced signalling pathways, IL-8, and interferon [46]. Although many questions remain, the "minimal inflammation" induced by hyperpermeability seems to be an interesting explanation of the pathophysiological background of IBS. In previous work by our group [47], higher plasma levels of IL-6, IL-8, resistin, and adiponectin were shown in IBS-D patients compared to controls, supporting the hypothesis of an inflammatory component in this functional GI disorder. Conversely, in the present paper, IBS-D patients with high somatization showed a significant increase in IL-8 and a parallel decrease in IL-10 concentrations [48]. IL-8 and the related cytokines are reported to be high in several tissues in the case of different pathological conditions (e.g., infection, inflammation, ischemia, trauma) and are thought to be able to induce a local neutrophil accumulation [49].

On the contrary, IL-10 is considered a potent negative feedback regulator that induces resolution of inflammation via autocrine and paracrine mechanisms [50]. The reduced levels of IL-10 expressed in IBS-D patients with HS are consistent with these properties.

In support of the alterations in the intestinal barrier of HS patients, also LPS concentrations were significantly higher in these patients than in LS ones. LPS is a surface molecule of many gram-negative bacteria, and high circulating levels suggest bacterial translocation [19]. Psychological disorders such as anxiety and depression appear to be associated with impaired intestinal permeability and dysbiosis with LPS-secreting bacteria in the plasma [19]. This hypothesis has recently been confirmed by a correlation between markers of increased intestinal permeability (zonulin and I-FABP), dysbiosis, and increased LPS concentrations in subjects with depression/anxiety compared to controls. These results highlight that evaluating the state of health of the gut can be considered a new target for diagnosing and managing mental health, even in patients not suffering from GI disorders [19].

Much attention has recently been paid to non-invasive methods for evaluating dysbiosis in the gut. The small intestine's bacterial proliferation has already been associated with the IBS-D subtype and is considered responsible for the onset of IBS symptoms. In our study, urinary indole concentrations, a marker of fermentative dysbiosis [51–53], were higher in patients with HS than LS ones, thus confirming the intestinal bacterial microbiota's alterations in IBS-D [54]. Further evidence of the central involvement in intestinal bacteria derives from probiotics in IBS management, as in the case of Bifidobacterium longum NCC3001 able to improve the IBS patient’s QoL while reducing the depression levels [55].

The present research has some weaknesses. First, the cohort of patients was too small to draw firm conclusions. However, based on present and other data in the literature, it seems conceivable that high somatization levels are tightly connected with alterations in the intestinal barrier and can determine massive effects on GI symptoms of IBS-D patients. Secondly, the inflammation markers were not evaluated in intestinal biopsies. Thus, we could not obtain complete information about the actual intestinal processes occurring at a mucosal level. Lastly, dysbiosis was investigated utilizing an indirect analysis instead of more robust and efficient methods such as evaluating sequences of bacterial 16S rRNA gene or its components that could have provided more detailed information on the positive effects of the diet.

## Conclusions

In conclusion, the identification of IBS should not be limited to Rome criteria but must be completed by the psychological profile.

Our HS patients showed a high symptoms profile and pathophysiological and biochemical modifications suggestive of altered intestinal barrier function and integrity, such as increased s-IP, inflammation, and intestinal fermentative dysbiosis. Therefore, IBS must be considered a multidimensional condition, needing in-depth and complete clinical, psychological, and biochemical evaluation.

## Data Availability

The datasets used and/or analysed during the current study are available from the corresponding author on reasonable request.

## Abbreviations

BMI: Body mass index
DAO: Diamine oxidase
EDTA: Ethylen-diamine-tetra-acetic
EMA: Anti-endomysium
GI: Gastrointestinal
GSI: Global score index
GSRS: Gastrointestinal Symptom Rating Scale
HC: Healthy controls
HS: High somatization
IBD: Inflammatory bowel disease
IBS: Irritable bowel syndrome
IBS-C: IBS with prevalent constipation
IBS-D: IBS with prevalent diarrhoea
IBS-M: Alternate/mixed IBS
IBS-SSS: IBS Severity Scoring System
IBS-U: Unclassified IBS
I-FABP: Intestinal fatty-acid binding protein
IL: Interleukins
La: Lactulose
LPS: Lipopolysaccharide
LS: Low somatization
Ma: Mannitol
SCL-90-R: Symptom Check list-90-Revised
S-IP: Small intestinal permeability
SSD: Somatic symptom disorder
SSRIs: Selective serotonin reuptake inhibitors
Su: Sucrose
TNF: Tumour necrosis factor
tTG: Tissue transglutaminase
